# Association among first-aid literacy, training and life meaning: a KAP study of first-year undergraduates at a comprehensive university in China

**DOI:** 10.3389/fpsyg.2026.1915728

**Published:** 2026-10-01

**Authors:** Yan Xu, Jia Li, Ying Jiang, Yichen Li, Tie Xu, Meijia Zhu, Jing Lin, Dianya Zhang, Zhaohui Qin

**Affiliations:** 1 Research Center for Medical and Health Emergency Rescue, School of Public Health, Xuzhou Medical University, Xuzhou, Jiangsu, China; 2 The Second Clinical Medical School, Xuzhou Medical University, Xuzhou, Jiangsu, China; 3 The First Clinical Medical School, Xuzhou Medical University, Xuzhou, Jiangsu, China

**Keywords:** first-aid literacy, first-year undergraduate, KAP, meaning in life, mediation analysis

## Abstract

**Background:**

First-aid education has been widely implemented in universities, with first-aid literacy serving as key evaluation indicators. Meaning in life (MIL) positively influences health awareness and prosocial behavior; however, its relationship with first-aid literacy remains insufficiently explored. This study investigated the association between meaning in life and safety and first-aid literacy among first-year undergraduate students and examined the mediating role of prior first-aid training times.

**Methods:**

A total of 4,723 first-year undergraduate students from a comprehensive university completed a social-factor questionnaire, the Meaning in Life Questionnaire, and the First-aid Literacy Questionnaire based on the Knowledge-Attitude-Practice model. After data cleaning, 4,708 valid responses were analyzed. Correlation and mediation analyses were conducted to assess relationships among variables.

**Results:**

Knowledge scores followed a left-skewed distribution, whereas skill scores were concentrated at moderate levels. MIL scores presented an overall upward distribution. Correlation analysis revealed significant associations among most variables (*p* < 0.05). No significant correlations were observed between first-aid skills and the two dimensions of meaning in life, nor between knowledge and prior training times. Mediation analysis suggested a statistically significant indirect association between MIL and first-aid skills through training times, whereas no significant indirect association was observed for first-aid knowledge. The association between MIL and attitude was mainly dominated by the direct effect.

**Conclusion:**

Integrating MIL into first-aid education may enhance students’ first-aid literacy. Promoting a balanced life-meaning orientation can improve training effectiveness, particularly with increased training exposure, supporting integrated psychological–health education models.

## 1 Introduction

### 1.1 Safety and first-aid literacy in universities

Safety and first-aid literacy, recognized as one of the six core components of health literacy in China (NHC P, 2016; NHC P, 2024), reflects individuals’ awareness of public safety as well as their capacity to perform self-rescue and provide mutual aid during emergency situations (Yan et al., 2015). Adequate public health and safety literacy is a fundamental prerequisite for effective participation in the prevention and control of public health incidents and constitutes an essential element of national efforts to strengthen systemic weaknesses and address existing gaps within the public health system (Ma, 2020). Enhancing safety literacy plays a pivotal role in promoting public health by reducing preventable injuries, improving emergency response capabilities, and reinforcing both individual and collective resilience to health-related risks. In recent years, the frequent occurrence of public emergencies has posed serious threats to social life and safety (Yang et al., 2009). Universities, characterized by high population density, are particularly vulnerable to public health incidents and safety accidents (Li, 2015). Most first-year undergraduate students are transitioning from adolescence to adulthood. They move from familiar environments to new campus settings, beginning long-term collective living and learning, and face unique health and safety risks distinct from those encountered previously or by other populations. This population urgently requires public health and safety literacy tailored to their developmental stage and the demands of prolonged communal living. Studies indicate that the first-year undergraduate student with strong fire safety awareness and first-aid knowledge are more likely to take protective actions and provide timely assistance to others, thereby contributing to overall campus safety (Wang et al., 2016).

### 1.2 Knowledge-attitude-practice model

The Safety and first-aid Literacy Questionnaire is a standardized instrument designed to assess residents’ knowledge and skills in safety and first-aid. The average score among Chinese residents is 6.13 out of a possible 10 points (NHC P, 2025), indicating substantial room for improvement in overall safety and first-aid literacy. However, based on previous evaluation experience, the assessment of an individual’s fundamental literacy typically involves a comprehensive consideration of knowledge, attitude, and practice, according to the knowledge-attitude-practice model (KAP). The KAP survey originated in the 1950s in the fields of family planning and population research. The model posits that knowledge serves as the foundation, attitude acts as the intermediary, and behavior (or skills) represents the ultimate manifestation, with all three dimensions collectively constituting “literacy.” In health education and behavioral intervention research, an individual’s fundamental literacy is not determined by a single dimension but is generally assessed across three levels: knowledge—the degree of mastery of relevant information; attitude—values, beliefs, and motivation; and practice—the actual behaviors and skill performance (Jacobsen, 2016).

In the field of safety and first-aid, possessing knowledge or skills alone is insufficient to ensure that individuals will take appropriate rescue actions in real-life situations; rescue-related beliefs, attitudes, and a sense of responsibility also play critical roles (Xie et al., 2024). Therefore, when evaluating students’ safety and first-aid literacy, it is essential not only to assess their knowledge level but also to consider their rescue-related beliefs and practical skills, in order to provide a more comprehensive reflection of their safety and first-aid literacy and ability to respond effectively to emergencies.

### 1.3 Training and safety and first-aid literacy

Multiple studies have demonstrated that first-aid training effectively enhances individuals’ safety and first-aid literacy. A recent review reported that, compared with untrained individuals, nonprofessional populations receiving first-aid training exhibited significant improvements in knowledge, practical skills, and self-efficacy, highlighting the effectiveness of such training in strengthening fundamental first-aid literacy (Kendall et al., 2025). Similarly, empirical studies have shown that educational interventions significantly improve both theoretical knowledge and practical skills, as evidenced by comparisons between pre-training and post-training assessments (Fernández-García et al., 2023). Furthermore, improvements in self-efficacy following training provide additional evidence that these interventions enhance individuals’ confidence in performing first-aid and rescue actions (Matsuyama et al., 2020). More recent evidence has broadened the concept of first-aid competence beyond technical proficiency alone. Studies among nursing students have demonstrated that psychological first-aid practice self-efficacy is a significant predictor of disaster response self-efficacy (Durmuş Sarıkahya et al., 2026), while disaster preparedness literacy is positively associated with psychological first-aid practice self-efficacy (Cinar Ozbay et al., 2026). Together, these findings suggest that first-aid competence extends beyond the acquisition of knowledge and practical skills, encompassing the confidence and preparedness necessary for effective emergency and disaster response. Consequently, comprehensive first-aid education should aim to cultivate not only technical competence but also the psychological readiness required to respond appropriately in emergency situations.

Therefore, assessing students’ safety and first-aid literacy is an essential prerequisite for designing targeted and effective first-aid training interventions. Such assessments can help identify specific educational needs and provide a foundation for developing training programs that ultimately improve students’ overall safety awareness, first-aid competence, and emergency preparedness.

### 1.4 The role of understanding the meaning of life

Current research on first-aid education in universities has primarily focused on sociological factors such as age, gender, and family background (Kwon and Chong, 2003; Ng et al., 2021; Reveruzzi et al., 2016), while largely overlooking the role of psychological development and life education. The neglect of life education has, in turn, led some college students to hold vague or distorted views of life, making it difficult for them to correctly understand the essence and value of life (Zhang et al., 2012). Research shows that universities often incorporate the Meaning in Life (MIL) psychological assessment tool in health education and mental health evaluations (Li and Jia, 2022). This scale assesses students’ cognitive levels regarding life and reflects their attitudes toward the value of their own and others’ lives, thus facilitating a deeper understanding of their behavioral motivations and psychological response patterns.

MIL, is a vital component of subjective well-being. It refers to an individual’s cognitive awareness of life purpose, existence value, and significance, represents an important psychological construct and is widely recognized as a key protective factor against suicidal behavior (Van Deurzen, 2001; Wong and Fry, 1998). Research indicates that individuals’ understanding of life meaning is multifaceted and highly variable, exhibiting significant dynamic characteristics influenced by cultural background, religious beliefs, personal differences, and life experiences (Frankl and Lasch, 1962). Furthermore, several studies have demonstrated that a greater sense of life meaning contributes to improved health-related quality of life (Aftab et al., 2019). Individuals with a strong sense of life meaning tend to adopt more positive coping strategies when facing stress and crises, exhibit greater self-efficacy, perform better in interpersonal relationships and social adaptation, and show a reduced risk of psychological problems (Yalçın and Malkoç, 2015). Accordingly, we propose that cognition of the meaning in life plays a critical role in shaping safety and first-aid literacy by influencing individuals’ safety-related attitudes and behaviors.

In 2006, Michael F. Steger and colleagues developed and published the meaning-in-life questionnaire (MLQ) (Steger et al., 2006). The MLQ conceptualizes MIL as comprising two dimensions: the presence of meaning in life (PML) and the search for meaning in life (SML). PML refers to an individual’s recognition of life’s significance, value, and purpose—that is, the extent to which one perceives meaning, goals, or a mission in life. SML reflects the active motivation to explore and enhance life’s meaning and purpose, encompassing efforts, intensity, and behaviors in this pursuit (Wang et al., 2018). The MLQ has demonstrated strong psychometric properties and has shown good applicability and reliability across different age groups (Schutte et al., 2023) and cultural contexts (Khumalo et al., 2014; Rose et al., 2016).

### 1.5 The present study

Previous studies have demonstrated that differences in the cognition of meaning in life can substantially influence individuals’ prosocial attitudes and behaviors, and that engagement in prosocial behaviors, in turn, can serve as an important source of perceived meaning in life (Klein, 2017; Van Tongeren et al., 2016). Although research on MIL is well established, and there is evidence suggesting that life meaning may influence individuals’ safety behaviors and awareness (Hu and Lü, 2022; Yildirim et al., 2024). However, its impact on college students’ safety and first-aid literacy has not been systematically examined. The integration of life ethics and meaning education into first-aid curricula remains unclear. Due to limited theoretical and practical guidance, effective first-aid education strategies are scarce. This study employs correlation matrix analysis of existing survey data to investigate the associations between the PML and SML dimensions of the MLQ and students’ safety and first-aid literacy. Additionally, we examine whether the number of prior first-aid trainings mediates the relationship between life meaning cognition and safety and first-aid literacy. The findings may inform the design of first-aid education programs and policy development in universities.

## 2 Materials and methods

### 2.1 Participants and procedure

In September 2024, a cross-sectional survey using a stratified cluster sampling design was conducted at a comprehensive university. Eligible participants comprised all first-year undergraduate students enrolled in the 2024 cohort. Students were stratified according to academic discipline, including natural sciences, engineering, economics-related majors, and humanities. Within each discipline, four majors were randomly sampled, and all classes within these majors were invited to participate. Data were collected through the Wenjuanxing^1^ online survey platform. Before the formal survey, a pilot study was performed to refine item presentation, identify potential errors and inconsistencies, and estimate questionnaire completion time. Of the 4,723 questionnaires collected, 15 invalid responses were excluded, yielding a final analytical sample of 4,708 participants and a valid response rate of 99.68%. The study population consisted primarily of engineering students (71.0%), followed by humanities (13.7%), natural sciences (10.6%), and economics (4.7%).

### 2.2 Measures

#### 2.2.1 Meaning in life

The PML and SML subscales of the Meaning in Life Questionnaire Short Form were used to assess meaning in life, used the Chinese-translated version of Michael F. Steger’s Meaning in Life Questionnaire (Wang, 2013). Each item was rated on a five-point Likert scale (0–4; strongly disagree to strongly agree), yielding a total score of 0–40. The scores for PML, SML, and total MIL were averaged over five items and categorized as very low, low, average, strong, or very strong. In this study, the MLQ demonstrated acceptable internal consistency, with a Cronbach’s *α* of 0.886.

#### 2.2.2 Previous first-aid training

We collected the students’ professional information, and we assessed whether participants had ever attended first-aid training, with five response options: 0, 1, 2, 3, or more than 3 sessions.

#### 2.2.3 Safety and first-aid literacy

The Safety and First-aid Literacy Questionnaire, was divided into three domains: knowledge, attitude, and skills. The knowledge domain primarily employed the standardized questionnaire from Part IV—Safety and First-aid Literacy—of the Chinese Residents’ Health Literacy Surveillance Report (Chinese Center for Health, 2022) comprising 10 items in total, with each item scored as 1 point, and the Cronbach’s *α* coefficient was 0.792, indicating acceptable reliability for use. The attitude domain assessed respondents’ willingness to provide first-aid when encountering an injured or ill person, measured using a five-point Likert scale. The skills domain consisted of 17 items evaluating competencies related primarily to cardiopulmonary resuscitation (CPR) and the use of automated external defibrillators (AED), with each item scored as 1 point. All items were derived from the professional examination item bank used for assessing licensed physicians in the field of emergency care. In this study, the Cronbach’s *α* coefficient was 0.835, indicating acceptable reliability for use.

## 3 Data analysis

Descriptive analyses were performed on data from the three questionnaires. The Shapiro–Wilk test indicated that some variables deviated from normality; therefore, nonparametric Spearman rank correlation, implemented in R Studio, was used to construct a correlation matrix. The matrix included two dimensions of meaning in life (PML and SML), three dimensions of safety and first-aid literacy (Knowledge, Attitude and Skills), and participants’ prior first-aid training times (Times). Bivariate relationships among these variables were examined, and correlation coefficients with corresponding significance levels (*p* < 0.05) were reported.

To further examine the relationships among meaning in life, prior first-aid training, and safety and first-aid literacy, we conducted a mediation analysis using prior first-aid training as the mediator. The quantified scores of the SML and PML dimensions of meaning in life served as independent variables, and the Knowledge, Attitude and Skills subscales of safety and first-aid literacy were the dependent variables.

Statistical analyses were performed in R Studio (R Foundation for Statistical Computing, Vienna, Austria). The haven and dplyr packages were used for data management. Correlation analyses and visualizations relied on rcompanion, corrplot, and RColorBrewer. Mediation analysis was implemented in lavaan, employing 5,000 bootstrap resamples to estimate indirect effects and derive bias-corrected and accelerated (BCa) 95% confidence intervals. Mediation effect forest plots were created using ggplot2, while pathway diagrams were produced via DiagrammeR. The mediation pathway is illustrated in Figure 1.

**Figure 1:**
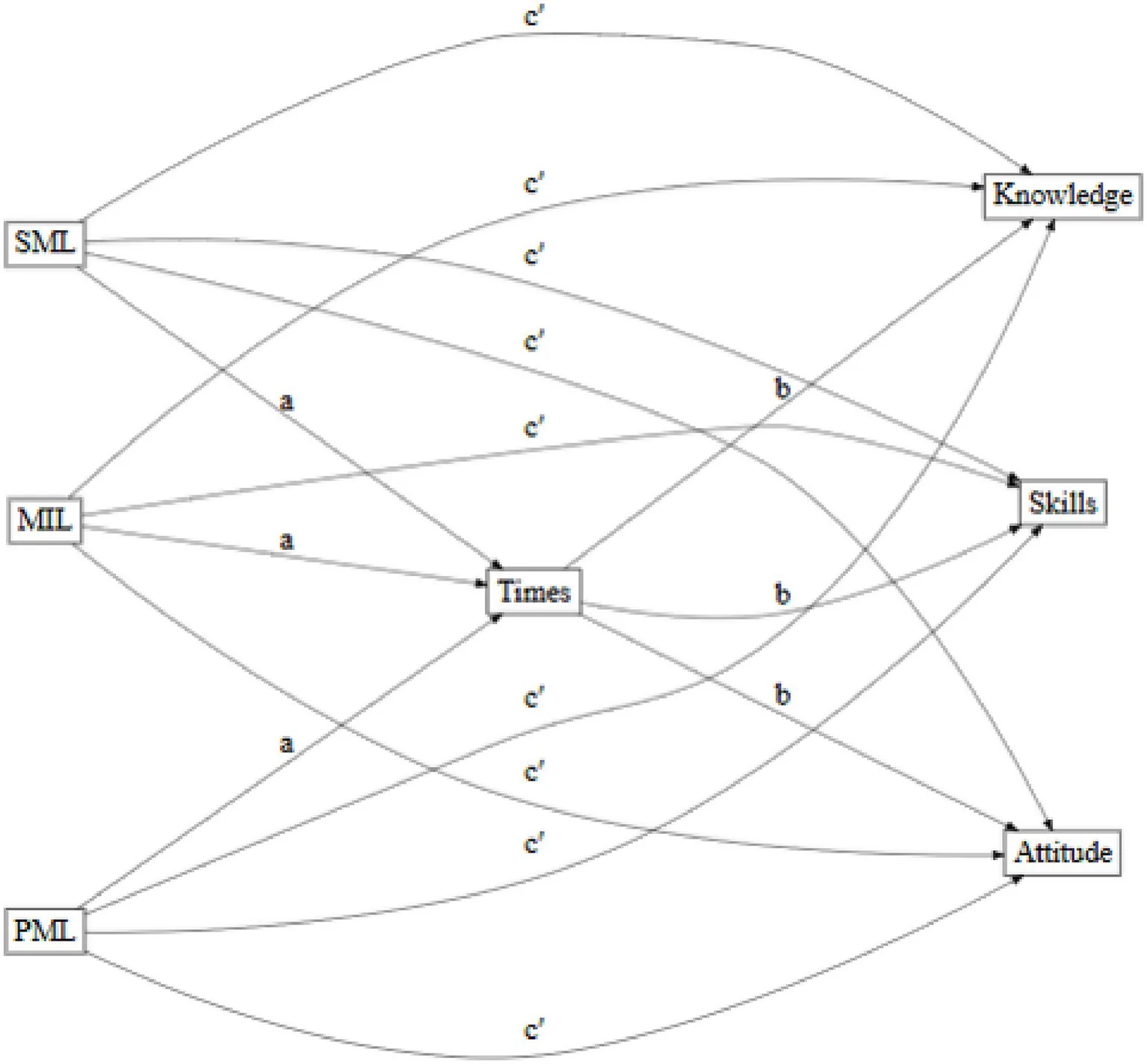
Diagram of the process flow for mediation effect path **(a)**: X → M; path **(b)**: M → Y (controlling for X); path **(c’)**: X → Y (direct effect, controlling for M).

Table 1 illustrates the overall distribution of independent and dependent variables. The average score of MIL was 29.28 ± 6.97, with a median of 29.00. The mean score for SML items was 15.78 ± 3.90 (median: 16.00), whereas the average score for the PML items was 13.51 ± 4.56 (median: 14.00). Both the total score and the SML subscale exhibited an increasing number of respondents with higher scores, whereas scores on the PML subscale were primarily concentrated at the moderate level.

**Table 1:** Statistical parameters of score-based questionnaire.

Score	SML	PML	MIL	Knowledge	Skills
AVG^a^	15.78	13.51	29.28	8.26	7.93
SD^b^	3.90	4.56	6.97	1.51	2.41
MED^c^	16.00	14.00	29.00	9.00	8.00

^a^AVG, average; ^b^SD, standard deviation; ^c^MED, median.

The mean score for safety and first-aid knowledge was 8.26 ± 1.51, with a median of 9.00, which were concentrated at 8 points (25.40%) and 9 points (32.37%). Its overall scores were clustered in the upper-middle range, with only a small proportion at the extremes, indicating a left-skewed distribution. The mean score for safety and first-aid skills was 7.93 ± 2.41, with a median of 8.00. The highest proportion of scores (19.41%) occurred at 8 points, and the distribution was approximately normal.

Non-quantitative indicators (willingness to provide rescue and training times) were quantified using a 5-point Likert scale, with scores coded sequentially as 0, 1, 2, 3, and 4. Raw questionnaire data are presented in Table 2: 33.9% of respondents reported having received relevant training, whereas 66.1% had not. In terms of participants’ attitudes, 79% of students exhibited moderate or higher willingness to deliver first aid.

**Table 2:** Results of non-quantitative items.

Option	Number	%	Option	Number	%
Previous number of training sessions			Willingness to rescue		
0	3,114	66.1	Poor	22	0.5
1	269	5.7	Fair	76	1.6
2	259	5.5	Average	888	18.9
3	479	10.2	Good	1,272	27.0
>3	587	12.5	Excellent	2,450	52.0

## 4 Results

### 4.1 Correlation matrix analysis

The detailed correlation coefficients matrices and and their significance are presented in Figure 2. The correlation matrix analysis indicated that Attitude exhibited significant moderate positive correlations with SML (*r* = 0.35), PML (*r* = 0.32), and overall MIL (*r* = 0.39). However, the correlations of Knowledge and Skills with the three meaning-in-life variables (MIL, PML, SML) were either generally weak (|*r*| < 0.20) or statistically non-significant.

**Figure 2:**
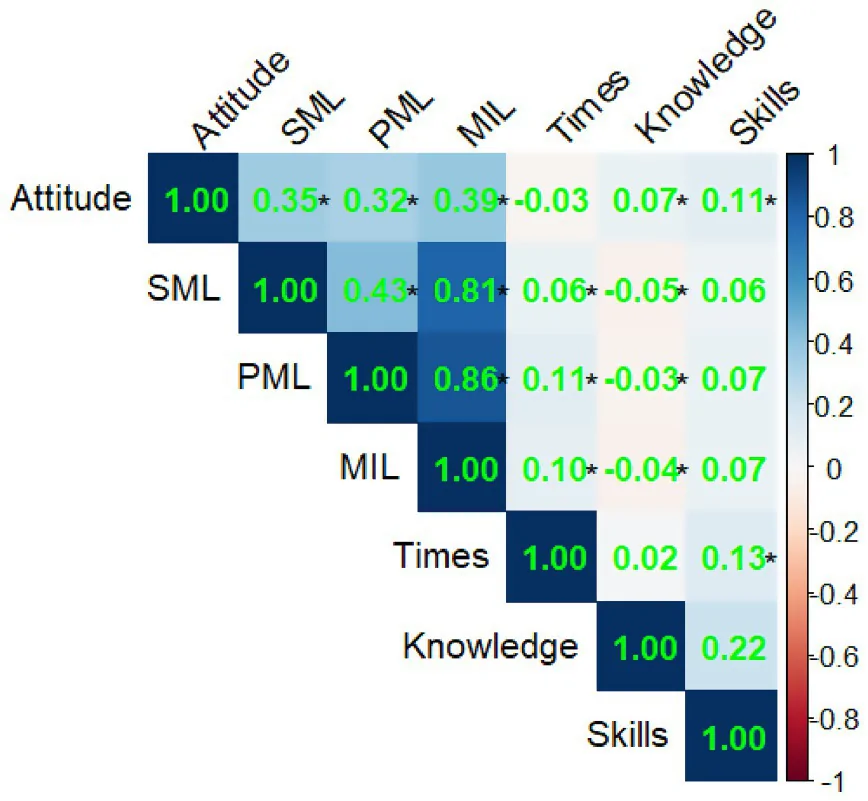
Correlation degree diagram of factors in the relevant matrix. The numbers in the figure represent correlation coefficients. A value closer to 0 indicates a lower correlation, with + representing a positive relationship and—a negative one. * indicates statistical significance at the level of *p* < 0.05.

Besides, the variable of Times was significantly positively correlated with MIL, SML, PML and Skills (*r* < 0.15). In addition, a relatively strong correlation was observed between SML and PML (*r* = 0.43). Simply summing SML and PML to derive the MIL total score presupposes that the two subscales jointly capture the construct of meaning in life, although the aggregation necessarily counts the component shared by the two dimensions twice. Given this overlap, the same total score may conceal qualitatively different subscale combinations: individuals with high PML and low SML may score similarly to those with low PML and high SML.

### 4.2 Mediation analysis

Table 3 reports the standardized path coefficients of the mediation effect test and the 95% BCa CIs of estimate coefficients, which demonstrate heterogeneous performances across the three dimensions of safety and first-aid literacy. For the first-aid attitude domain, MIL, PML and SML yielded significant direct effects and indirect effects through training times (all *p* < 0.05). Specifically, the indirect effect was significantly negative (|a × b| < 0.01), whereas the direct effect was significantly positive (c’ > 0.3). In contrast, no significant direct, indirect, or total effects were detected for first-aid knowledge (all *p* > 0.05). Regarding first-aid skills, significant indirect effects through training times were observed for MIL (standardized coefficients a × b = 0.011, *p* < 0.05), PML (std. a × b = 0.012, *p* < 0.05), and SML (std. a × b = 0.005, *p* < 0.05), while the corresponding direct and total effects remained non-significant (all *p* > 0.05). These results indicated that training times served as a crucial mediating variable in the association between meaning in life and first-aid skills, whereas such a mediating pathway was not identified for first-aid knowledge.

**Table 3:** Estimated parameters and confidence intervals of the mediation effect.

Paths & parameters	Standardized coefficients	Estimate coefficients	95% BCa CIs		Paths and parameters	Standardized coefficients	Estimate coefficients	95% BCa CIs	
			Lower	Upper				Lower	Upper
MIL→Knowledge					PML → Knowledge				
a	0.108	0.025*	0.019	0.032	a	0.120	0.043*	0.033	0.053
b	0.006	0.006	−0.022	0.032	b	0.006	0.006	−0.022	0.032
c’	−0.031	−0.007	−0.014	0.001	c’	−0.027	−0.009	−0.019	0.001
a × b	0.001	0.000	−0.001	0.001	a × b	0.001	0.000	−0.001	0.001
c	−0.030	−0.007	−0.014	0.001	c	−0.026	−0.009	−0.018	0.001
MIL→Skills					PML → Skills				
a	0.108	0.025*	0.019	0.032	a	0.120	0.043*	0.033	0.053
b	0.100	0.148*	0.107	0.195	b	0.100	0.149*	0.107	0.196
c’	−0.015	−0.005	−0.015	0.005	c’	−0.017	−0.009	−0.024	0.005
a × b	0.011	0.004*	0.002	0.005	a × b	0.012	0.006*	0.004	0.009
c	−0.004	−0.002	−0.012	0.008	c	−0.005	−0.003	−0.018	0.012
MIL→Attitude					PML → Attitude				
a	0.108	0.025*	0.019	0.032	a	0.120	0.043*	0.033	0.053
b	−0.058	−0.028*	−0.040	−0.015	b	−0.053	−0.025*	−0.038	−0.013
c’	0.380	0.042*	0.039	0.045	c’	0.302	0.051*	0.047	0.056
a × b	−0.006	−0.001*	−0.001	0.000	a × b	−0.006	−0.001*	−0.002	−0.001
c	0.374	0.042*	0.039	0.045	c	0.296	0.050*	0.046	0.055
SML → Knowledge					SML → Skills				
a	0.053	0.022*	0.010	0.034	a	0.053	0.022*	0.010	0.034
b	0.004	0.004	−0.024	0.030	b	0.099	0.147*	0.105	0.191
c’	−0.023	−0.009	−0.021	0.004	c’	−0.007	−0.004	−0.022	0.013
a × b	0.000	0.000	−0.001	0.001	a × b	0.005	0.003*	0.002	0.006
c	−0.023	−0.009	−0.021	0.004	c	−0.002	−0.001	−0.019	0.017
SML → attitude									
a	0.053	0.022*	0.010	0.034					
b	−0.034	−0.016*	−0.029	−0.003					
c’	0.324	0.064*	0.058	0.071					
a × b	−0.002	0.000*	−0.001	0.000					
c	0.322	0.064*	0.058	0.070					

^a^* indicates statistical significance at the level of *p* < 0.05; ^b^Parameters a, b, c’ denote the path coefficients for the independent variable on the mediator, the mediator on the dependent variable, and the independent variable on the dependent variable, respectively; ^c^Parameters a × b and c denote the indirect mediation effect and total effect, respectively.

Consistent with the above findings, the BCa 95% bootstrap confidence intervals further validated the mediation results (Figure 3). Specifically, the confidence intervals for significant indirect effects did not include zero, whereas the intervals for all non-significant effects spanned zero, confirming the robustness of the observed mediation patterns.

**Figure 3:**
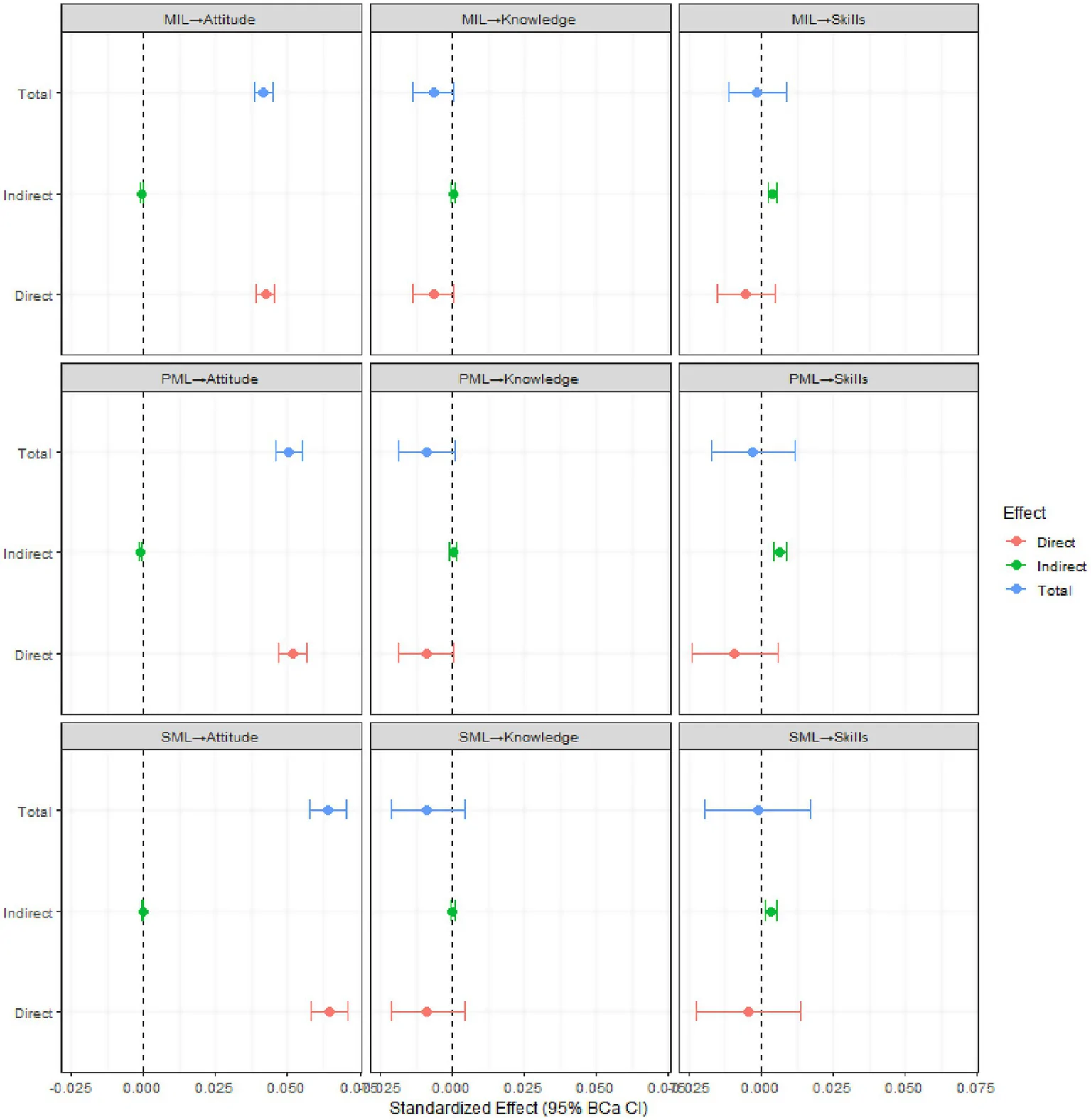
Forest plots of standardized total, direct, and indirect effects of meaning in life on safety and first-aid literacy outcomes (95%BCa). The vertical dashed line denotes a standardized effect of zero; statistical significance was established when the 95% CIs did not span zero. Overall, MIL, PML, and SML exhibited consistent positive associations with first-aid attitude, with both total and direct effects significantly different from zero.

Collectively, the present results reveal that meaning in life exerts domain-specific differential influences on first-aid literacy: it strongly and positively shapes first-aid attitudes, exhibits negligible associations with first-aid knowledge, and improves first-aid skills indirectly via engagement in first-aid training.

## 5 Discussion

This study aimed to investigate the relationships between first-year undergraduate students’ understanding of MIL and their safety and first-aid literacy, with a particular focus on the three components of literacy: knowledge, attitude, and practice. Under the unified measurement of the Safety and First Aid Knowledge Literacy Questionnaire, first-year undergraduates in the present study exhibited an overall higher level of safety and first aid knowledge literacy compared with the national average level. This result may be associated with China’s current social stability and the relatively high levels of well-being observed among students (Lai et al., 2019). Nevertheless, the safety and first-aid literacy questionnaire assesses only the most fundamental safety-related knowledge, and an optimal level would be expected to exceed 9 points, indicating considerable potential for further enhancement.

### 5.1 Association between meaning in life and safety and first-aid literacy

The correlation matrix analysis showed that MIL was very weakly associated with safety and first-aid knowledge and skills, but was strongly and significantly associated with rescue-related attitude. This pattern may be explained by the fact that MIL primarily influences individuals’ value orientation and prosocial motivation (Fu et al., 2022), thereby shaping behavioral attitude rather than directly enhancing knowledge or technical skills (Feng et al., 2025). A stronger sense of MIL has been shown to significantly increase intrinsic motivation, a sense of purpose, altruistic tendencies, and self-transcendence, which may motivate individuals to engage more readily in helping behaviors during emergencies, resulting in a stronger positive association with willingness to provide rescue assistance (Lv et al., 2024). In particular, individuals who are actively searching for MIL may be more willing to incur higher prosocial costs and demonstrate stronger rescue-related beliefs (Dakin et al., 2021). In contrast, the acquisition of knowledge and skills typically depends on external factors such as formal education, self-directed learning, and practical experience (Darling-Hammond et al., 2020). Because these factors are only indirectly related to MIL, the influence of meaning in life on knowledge and skills is correspondingly weak.

### 5.2 Mediating role of first-aid training times

According to the above analyses, MIL (including SML and PML) was consistently positively associated with favorable first-aid attitude. Mediation analyses further confirmed partial mediation between MIL and first-aid attitude. However, the direct effect predominated in this relationship, as the indirect effect had a small negative coefficient. This observation indicates that, relative to theoretical first-aid knowledge and practical skills, people’s attitudes toward first aid show stronger correlational links with purpose in life and prosocial values; training times exhibits only a modest mediating association within this relational pathway.

For first-aid skills, the absolute magnitude of the coefficient for the non-significant negative direct association slightly surpassed that of the significant positive indirect association. The offsetting nature of these two associations produced a suppression pattern, with their aggregate net association close to zero. These results imply that MIL exhibits limited capacity to relate to first-aid skills via a pathway separate from the mediator, and training times constitutes a relevant mediating relational pathway. Inclusion of this mediating variable helps disentangle the underlying relational mechanism, illustrating the analytical value of mediation analysis.

In contrast, training times exhibited no significant indirect association connecting MIL and first-aid knowledge. The differing mediating relational patterns of training times for first-aid knowledge versus first-aid skills imply that first-aid knowledge is primarily gained through cognitive learning and information processing, whereas first-aid skills are more closely linked to practical experience and hands-on training (Mahony et al., 2008). Existing studies note that knowledge declines more slowly than practical skills post-training (Anderson et al., 2011), and skill retention correlates with repeated practice and continuous training exposure (Zenani et al., 2022). Consistent with prior work, more frequent first-aid training participation correlates with greater skill proficiency and sustained skill retention (Anderson et al., 2011). In comparison, knowledge acquisition relies more on the quality of initial learning; inadequate initial learning may coincide with poorer long-term knowledge retention notwithstanding follow-up training (Eppinger et al., 2011).

In addition, the significant correlations of training times with MIL and first-aid skills revealed in the mediation analysis indicate that individuals with higher levels of MIL may be more willing to participate in first-aid training and may report stronger prosocial motivation and better rescue skills (Dakin et al., 2021).

### 5.3 Findings regarding the mechanisms of meaning in life

We observed a significant positive correlation between the SML and PML dimensions, indicating that these two constructs are not entirely independent, a finding consistent with previous studies (Naghiyaee et al., 2020; Newman et al., 2018). The search for meaning does not necessarily imply a lack of meaning. Prior research suggests that, within certain populations and developmental stages, the process of meaning exploration may mutually reinforce the formation and consolidation of meaning presence (Steger et al., 2008; Yek et al., 2017). Cross-cultural studies have further demonstrated that, among young adults, search for meaning and presence of meaning are often positively correlated. From the perspective of meaning-making theory, searching for meaning represents a critical pathway through which individuals continually affirm and strengthen their sense of life meaning (Park, 2010). Additionally, from the perspective of self-determination theory, a higher level of meaning presence is often accompanied by greater autonomy and intrinsic motivation, which in turn facilitates continued engagement in meaning exploration (Aftab et al., 2019).

### 5.4 The role of safety and first-aid education practice

Safety and first-aid education is an effective approach to improving students’ safety and first-aid literacy, and incorporating MIL into such education may enhance training outcomes through multiple pathways. First, a stronger sense of MIL can increase intrinsic motivation and autonomy, thereby encouraging more active engagement in learning both knowledge and skills (Ryan and Deci, 2000; Tang et al., 2021). Second, MIL may promote prosocial tendencies and a sense of responsibility, increasing individuals’ willingness to provide assistance in emergency situations (Dakin et al., 2021). In addition, as a deep-seated value orientation, MIL promotes consistency between values and behavior, thereby improving learning attitude and practical engagement. In educational practice, integrating value-based discussions, case reflections, and exploration-oriented training into first-aid curricula may organically link MIL with skill acquisition, forming a closed loop of “meaning→attitude→ skills” and ultimately enhancing training effectiveness.

At the same time, it is important to guide and calibrate students’ orientation toward MIL. The goal is not to maximize meaning orientation indiscriminately but to provide appropriate guidance and correction. Insufficient meaning orientation may weaken individuals’ sense of responsibility and willingness to participate, whereas excessively high levels of meaning presence may inhibit further learning engagement and skill development (Kruger and Dunning, 1999). Therefore, safety and first-aid education should emphasize a dynamic balance between meaning presence and meaning search, guiding students to maintain value commitment while continuing reflection, learning, and capacity building.

The present findings provide important practical implications for higher-education first-aid education. Instead of offering fragmented, standalone first-aid training, universities should integrate disaster preparedness, psychological first aid, and emergency response training into undergraduate curricula within a structured, competency-based educational framework (Movahed et al., 2022). Specifically, first-aid literacy training can be embedded within general education and public health courses, supplemented by scenario-based simulations, multidisciplinary emergency drills, and community service-learning programs to strengthen students’ practical emergency competencies. Furthermore, regular refresher training and standardized competency assessments can effectively consolidate knowledge retention and sustain practical skill proficiency. Recent empirical evidence has validated that experiential and simulation-based pedagogies significantly enhance students’ emergency preparedness, situational decision-making, teamwork ability, and emergency response confidence. Collectively, these findings strongly support the adoption of a comprehensive, multi-dimensional first-aid education model in university settings (Baetzner et al., 2022; Mohamed et al., 2024).

## 6 Limitations

This study suggests that improving students’ safety and first-aid literacy may benefit from MIL education, which emphasizes goal setting and purpose. However, given the cross-sectional design, these findings should be interpreted as statistical associations rather than evidence of causal relationships. Future longitudinal and intervention studies are warranted to further examine the temporal relationships among meaning in life, first-aid training, and first-aid literacy.

Such education is often under-emphasized in practice, and there is a lack of systematic, evidence-based interventions. Our findings further suggest that other, stronger influencing factors—such as depressive symptoms—may also play a role in behavior change. Although existing evidence linking meaning in life to behavioral change remains limited, previous studies have demonstrated that meaning in life is associated with depression (Chen et al., 2021).

The study sample was drawn from newly enrolled university undergraduate students with relatively high educational levels. As such, the generalizability of these findings may be limited and may not reflect broader adolescent populations. Future research could explore interventions among more diverse youth populations and assess changes in health literacy following meaning-oriented education. However, such interventions may raise concerns about cost and resource allocation (Schippers and Hogenes, 2011). Balancing intervention reach with efficiency will be an important issue for future studies.

Several limitations remain in this study design. First, although stratified cluster sampling was employed, all participants were drawn from a single comprehensive university; such a single-institution sampling frame may limit the generalization of the findings to student populations at other colleges and universities. Second, all data were collected through self-administered questionnaires, which are inherently susceptible to social desirability bias and recall bias; participants may have over or under-reported certain behaviors or attitudes, thereby inflating or attenuating the observed associations among variables. Third, a cross-sectional design was adopted, which permits only the identification of correlational relationships among variables. Future longitudinal and experimental studies are warranted to further examine the causal pathways and temporal dynamics underlying these relationships.

## 7 Conclusion

This study provides preliminary evidence that integrating MIL concepts into first-aid education is both theoretically sound and practically feasible. The findings elucidate the mechanisms through which students’ perceptions of life’s meaning influence their safety and first-aid literacy. Specifically, the observed associations imply that fostering a balanced, constructive outlook on life meaning among students may correspond to improved outcomes from safety and first-aid training, with greater training exposure. These findings provide theoretical grounding and practical implications for designing integrated educational frameworks that merge psychological and health education.

## Data Availability

All data provided shall only be utilized for non-commercial academic research. Commercial application, secondary forwarding and distribution, re-identification of relevant subjects, as well as data alteration for commercial gains are all strictly forbidden. Requests to access the datasets should be directed to ZQ, qzh@xzhmu.edu.cn.

## Footnotes

1
https://www.wjx.cn/
